# Lost Working Years Due to Mental Disorders: An Analysis of the Norwegian Disability Pension Registry

**DOI:** 10.1371/journal.pone.0042567

**Published:** 2012-08-15

**Authors:** Ann Kristin Knudsen, Simon Øverland, Matthew Hotopf, Arnstein Mykletun

**Affiliations:** 1 Department of Health Promotion and Development, Faculty of Psychology, University of Bergen, Bergen, Norway; 2 Division of Mental Health, Department of Public Mental Health, Norwegian Institute of Public Health, Bergen, Norway; 3 Institute of Psychiatry, King's College London, London, United Kingdom; 4 School of Psychiatry, Faculty of Medicine, University of New South Wales, New South Wales, Australia; Federal University of Rio de Janeiro, Brazil

## Abstract

**Objectives:**

Mental disorders are prevalent diagnoses in disability benefit statistics, with awards often granted at younger age than for other diagnoses. We aimed to compare the number of lost working years following disability benefit award for mental disorders versus other diagnostic groups.

**Methods:**

Data from the complete Norwegian official registry over disability benefit incidence, including primary diagnoses, were analyzed for the period 2001 to 2003 (N = 77,067), a time-period without any reform in the disability benefit scheme. Lost working years due to disability benefit award before scheduled age retirement at age 67 were calculated.

**Results:**

Musculoskeletal disorders were the commonest reason for disability benefit awards (36.3%) with mental disorders in second place (24.0%). However, mental disorders were responsible for the most working years lost (33.8%) compared with musculoskeletal disorders (29.4%). Individuals awarded disability benefit for a mental disorder were on average 8.9 years younger (46.1 years) than individuals awarded for a musculoskeletal disorder (55.0 years), and 6.9 years younger than individuals awarded for any other somatic disorder (53.0 years). Anxiety and depressive disorders were the largest contributors to lost working years within mental disorders.

**Conclusion:**

Age at award is highly relevant when the total burden of different diagnoses on disability benefits is considered. There is great disparity in total number of lost working years due to disability benefit award for different diagnostic groups. The high number of lost working years from mental disorders has serious consequences for both the individual and for the wider society and economy.

## Introduction

Early work-life exit due to ill health is, besides being a potentially disastrous outcome for the individual, a social and economic challenge for developed economies [1]. Mental disorders are among the most prevalent diagnoses stated in disability benefit (DB) applications, and counts for an average of one third of new DBs awarded in the OECD countries [1]. Such high prevalence rates illustrate one aspect of the impact of mental disorders within DBs. The rate of DB award increase strongly with age [2], with most DBs awarded within a few years before the scheduled age of retirement. The age distribution varies across diagnostic groups, and there are indications that DBs for a mental disorder are generally awarded at younger age than DBs for other disorders [1], [3], [4]. The younger age at DB award for a mental disorder indicates that this diagnostic group is an important contributor to lost working years in the population.

Mental disorders constitute a heterogeneous diagnostic group, with variation in age of onset and functional impairment associated with the different classes of mental disorder. Mental retardation, disorders of psychological development (including learning disabilities and autism spectrum disorders) and emotional and behavioral disorders with onset in childhood or adolescence (i.e. attention deficit disorder) are often lifelong conditions which, when severe, may prevent the individual ever entering the workforce. Psychotic disorders typically have onset in the 20's to 30's [5] and are often associated with extensive functional impairment and stigma, which may make work participation in a competitive job market difficult [6]. For many of these individuals, DBs will probably be awarded early in working-age. Severe mental disorders are, however, much less prevalent than the common mental disorders anxiety and depression, both in the general population [7], [8], [9], [10] and within the DB statistics [4]. Anxiety and depression may impact on an individual throughout working life, and the age of DB award is likely to be between that of severe mental disorders and common somatic disorders, such as musculoskeletal disorders, cardiovascular disorders and cancer, which usually have their onset in late working-age.

The number of lost working years due to DB award may thus differ between diagnostic groups and classes, and may give figures that not necessarily correspond with prevalence estimates. It is thus important to go behind crude prevalence numbers from official DB statistics, and examine whether there are age differences between the diagnostic groups in regard to when they are awarded DB. If DBs for common mental disorders are awarded at a younger age compared with DBs for musculoskeletal or somatic disorders, this would add to what we know about the burden of these disorders in the community.

In the current study, using diagnostic information from the official Norwegian registry on permanent DB (disability pension) awards, we aimed to quantify lost working years associated with different diagnostic groups. Special attention was given to establish which classes within mental disorders that were causing the highest number of lost working years.

## Methods

### The disability benefit scheme in Norway and the FD-Trygd database

The Norwegian Social Insurance Scheme ensures income for individuals aged 18 to 66 who have had their working capacity permanently reduced by ≥50% due to illness, disease, injury, or disability accepted as a medical condition, and where there is little or no chance of future improvement of the working capacity. DB is paid until age 67, when the recipient is transferred to age-retirement pension. DB may be awarded to individuals who due to ill health never have been in paid work, but is not to be given for social problems like unemployment. It is further a prerequisite that the individual has attempted treatment and rehabilitation to improve the working capacity. The magnitude of the DB compensation depends on previous income, economical supporting responsibilities (i.e. children or spouse who cannot support themselves), and years of active work participation. In case of partial disability, for instance if the individual is capable to work 50%, the benefits are reduced correspondingly [11]. DB recipients may also earn a small income beside the benefits. The Norwegian disability benefit scheme is considered to be generous compared with DB schemes in other western countries [12].

FD-Trygd (Forløpsdatabasen Trygd) is the Norwegian national database with records on payment of state benefits to individuals within the Norwegian Social Insurance Scheme. The registry was established in 2000 and contains complete records from 1992 and onwards with continuous updates for individuals who receive DB. Statistics Norway administers the registry, and the data sources are administrative registries from Statistics Norway and the Norwegian Labor and Welfare Administration [13]. In the current study, information about new permanent DBs (disability pensions) awarded in the period 2001 to 2003 were employed, a period without any major reforms in the Norwegian DB scheme.

### Diagnostic information

FD-Trygd contains information about the primary diagnosis as presented in the application for a DB, coded according to the International Classification of Diseases (ICD) version 9 and 10 [14], [15]. Since 1998, the diagnostic information was coded in accordance with ICD-10.

We used three levels of categorization of diagnostic information; i) *main diagnostic groups* (mental disorders, musculoskeletal disorders and any other somatic disorder), ii) *diagnostic chapters* and iii) *classes within mental disorders*. The *diagnostic chapters* were defined in accordance with the diagnostic chapters in ICD-10. Seven of the ICD-10 diagnostic chapters constituted a total of 87.1% of the DBs awarded in the period 2001 to 2003, and were used as individual chapters. Diagnostic information was missing in 2.9% of the cases. The remaining 10.0% of cases were combined in an “other” category. *Classes within mental disorders* were also defined in accordance with their ICD-10 codes. All classes of mental disorders were included in the current study.

### Statistical analyses

*Years of working lost* were calculated by subtracting age when DB was awarded from age 67, which is the scheduled retirement age in Norway. Descriptive analyses were employed to examine total incidence, gender distribution, age at DB award, total lost working years and average number of lost working years within the three diagnostic levels. The results are presented both in descriptive tables and as line and bar graphs. As the results are based on complete records of DBs for the entire Norwegian population, no confidence intervals have been calculated. All analyses were conducted using STATA 11.0 [16].

### Ethics

As the information in FD-Trygd consist of routinely collected data which are anonymized and not possible to trace back to individuals, ethical approval from the regional ethics committee was not required for the current study.

## Results

**Figure 1:**
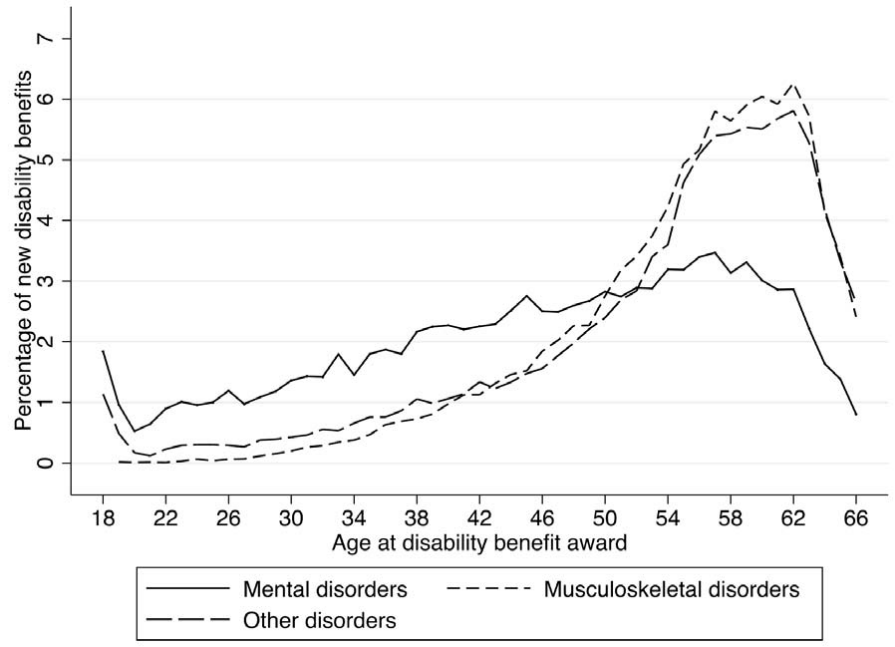
Age-distribution disability benefit award by diagnosis. Age-distribution of when disability benefits are awarded for mental disorders, musculoskeletal disorders and other somatic disorders. New permanent disability benefit awarded in Norway from 2001 to 2003.

**Figure 2:**
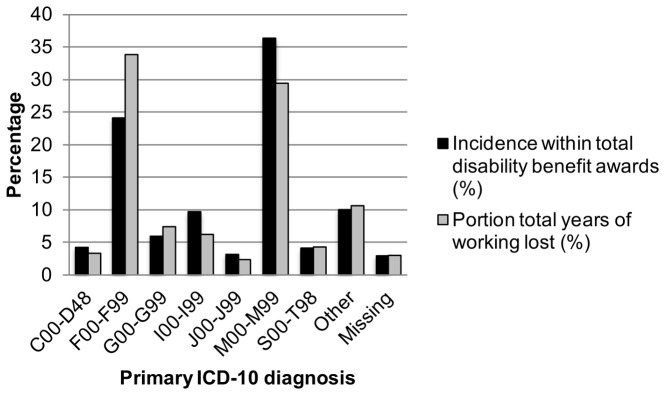
Incidence disability benefit award and lost working years by diagnosis. Proportions of total years of working lost and disability benefit award incidence per ICD-10 diagnostic chapter. New permanent disability benefits awarded in Norway from 2001 to 2003.

In the years 2001 to 2003 77,067 new DBs were awarded in Norway, which equals an incidence of 9/1000/year of the working-age population. A DB for a mental disorder was on average awarded 8.9 years earlier (mean: 46.1 years, standard deviation (SD): 12.4) than an award for a musculoskeletal disorder (mean: 55.0 years, SD: 7.9), and 6.9 years younger than any other somatic disorder (mean: 53.0 years, SD: 10.5) (Table 1 and Figure 1). Both musculoskeletal disorders and any other somatic disorder followed a trend with a steep increase in DB awards after the age of 50. In contrast, mental disorders had a more gradual increase in awards across the entire working-age span (Figure 1). Musculoskeletal disorders constituted the largest diagnostic group of all DB awards, with 36.3%, followed by mental disorders with 24.0% (Figure 2 and Table 1). However, when age at DB award was taken into account, mental disorders caused both the highest total and average number of lost working years compared with all other diagnostic groups, assuming age retirement at age 67 (Table 1). Awarded DBs for mental disorders in the period 2001 to 2003 gave in total 386,826 lost working years, equivalent to 33.8% of all lost working years, with an average of 20.9 (SD: 12.4) lost working years per recipient (Table 1, Figure 2). In comparison, DB award for musculoskeletal disorders caused 336,524 lost working years (29.4% of total lost working years, Figure 2), with an average of 12.0 (SD: 7.9) per recipient (Table 1, Figure 2).

**Table 1:** Distribution of disability benefits, mean age at award, years of working lost and percentage of women within ICD-10 diagnostic chapter.

Diagnostic chapter ICD-10	Prevalence^1^		Age^2^	Years of working lost^3^			Women
	No.	%	Mean (SD)	No.	Mean (SD)	%^4^	%
**C00-D48**Neoplasms	3,244	4.2	55.4 (7.9)	37,630	11.6 (7.9)	3.3	60.7
**F00-F99**Mental and behavioral disorders	18,505	24.0	46.1 (12.4)	386,826	20.9 (12.4)	33.8	52.9
**G00-G99**Diseases of the nervous system	4,523	5.9	48.2 (12.6)	84,852	18.8 (12.6)	7.4	52.6
**I00-I99**Diseases of the circulatory system	7,400	9.6	57.5 (6.7)	70,495	9.5 (6.7)	6.2	30.4
**J00-J99**Diseases of the respiratory system	2,358	3.1	56.0 (7.4)	25,917	11.0 (7.4)	2.3	46.9
**M00-M99**Diseases of the musculoskeletal system and connective tissue	27,994	36.3	55.0 (7.9)	336,524	12.0 (7.9)	29.4	62.6
**S00-T98**Injury, poisoning and certain other consequences of external causes	3,081	4.0	51.2 (10.6)	48,747	15.8 (10.6)	4.3	42.5
**Other**	7,726	10.0	51.3 (11.8)	121,140	15.7 (11.8)	10.6	54.4
**Missing**	2,236	2.9	51.8 (11.1)	33,983	15.2 (11.1)	3.0	48.6
Total	77,067	100.0	52.1 (10.8)	1,146,114	14.9 (10.8)	100.0	54.0

1 Prevalence within total number of permanent disability benefits.

2 Age at permanent disability benefit award.

3 Assuming age retirement at age 67.

4 Percentage within total lost working years.

As described in Table 2, developmental disorders (ICD-10 codes F80–F89, F90–F98) and mental retardation (ICD-10 codes F70–F79) had the highest average number of lost working years within mental disorders. However, due to their higher prevalence depressive disorders (ICD-10 codes F30–F39) and anxiety disorders (ICD-10 codes F40–F48) were responsible for the highest total numbers of lost working years, with 86,300 and 109,847 years respectively (Table 2).

**Table 2:** Distribution of disability benefits, mean age at award, years of working lost and percentage of women within the ICD-10 mental disorders chapter.

Class within mental disorders ICD-10	Prevalence			Age	Years of working lost^1^				Women
	No.	%^2^	%^3^	Mean (SD)	No.	Mean (SD)	%^4^	%^5^	%
**F00-F09**Organic, including symptomatic, mental disorders	480	2.6	0.6	48.6 (12.0)	8,820	18.4 (12.0)	2.3	0.8	38.1
**F10-F19**Mental and behavioral disorders due to psychoactive substance use	1,059	5.7	1.4	46.6 (9.3)	21,588	20.4 (9.3)	5.6	1.9	22.1
**F20-F29**Schizophrenia, schizotypal and delusional disorders	1,407	7.6	1.8	36.3 (10.9)	43,161	30.7 (10.9)	11.2	3.8	41.7
**F30-F39**Mood [affective] disorders	5,653	30.6	7.3	51.7 (9.7)	86,300	15.3 (9.7)	22.3	7.5	58.8
**F40-F48**Neurotic, stress-related and somatoform disorders	6,186	33.4	8.0	49.2 (9.9)	109,847	17.8 (9.9)	28.4	9.6	60.7
**F50-F59**Behavioral syndromes associated with physiological disturbances and physical factors	90	0.5	0.1	41.1 (11.1)	2,335	25.9 (11.1)	0.6	0.2	85.6
**F60-F69**Disorders of adult personality and behaviour	1,737	9.4	2.3	40.7 (9.7)	45,718	26.3 (9.7)	11.8	4.0	45.3
**F70-F79**Mental retardation	832	4.5	1.1	26.2 (10.6)	33,968	40.8 (10.6)	8.8	3.0	49.0
**F80-F89**Disorders of psychological development	368	2.0	0.5	25.8 (10.4)	15,174	41.2 (10.4)	3.9	1.3	36.4
**F90-F98**Behavioral and emotional disorders with onset usually occurring in childhood and adolescence	306	1.7	0.4	30.4 (10.2)	11,185	36.6 (10.2)	2.9	1.0	33.0
**F99**Unspecified mental disorder	387	2.1	0.5	44.4 (11.9)	8,730	22.6 (11.9)	2.3	0.8	50.9

1 Assuming age retirement at age 67.

2 Prevalence within permanent disability benefits awarded for mental disorders only.

3 Prevalence within total number of permanent disability benefits.

4 Percentage within years of working lost due to mental disorders.

5 Percentage within total years of working lost.

## Discussion

In this study, using complete records from the official Norwegian database over DBs awarded in the period 2001 to 2003, mental disorders were found to be the second most common diagnostic group within new DB awards, after musculoskeletal disorders. DBs for mental disorders were, however, awarded at a younger age than for all other disorders and conditions, which resulted in mental disorders causing the highest number of lost working years. Within mental disorders, developmental disorders and mental retardation had the highest average number of lost working years, whilst anxiety and depressive disorders constituted the highest total number of lost working years.

### Strengths and limitations

The main strength of this study is the completeness of the data. Being an official registry, FD-Trygd contains complete information of all new DBs awarded in Norway in the study's time-period. As correct registration in the DB registry is a prerequisite for transfer of DB payments, the records are highly reliable. The registry is continuously updated, which ensures that misclassifications, when discovered, are corrected.

FD-Trygd has good reliability in terms of whether the person is receiving DB or not, however, the validity of the primary diagnosis as an indication of the underlying medical condition causing the work disability is less certain. This uncertainty is potentially problematic if one is using the primary diagnoses in an attempt to quantify the impact of a disorder, or group of disorders, on the overall burden of DBs. The diagnostic information in FD-Trygd is based on the primary diagnosis stated on the DB application, usually given by the applicant's general practitioner, and the accuracy of the primary diagnoses will probably vary according to the category of illness. For primary diagnoses with clear biomedical diagnostic features, such as cancer, it is likely that the diagnosis will be accurate and will take precedence over comorbid conditions – it will therefore have high sensitivity and specificity, meaning that the impact of cancer on DB awards is probably almost completely ascertained. However, many categories of somatic disorder are less clear-cut. Within musculoskeletal disorders for instance, whilst some cases may include defined diagnoses such as rheumatoid arthritis, the majority of awards are for more amorphous and less well defined symptom-based conditions such as fibromyalgia, back pain and so on. For mental disorders, the lack of diagnostic precision is compounded both by comorbidity with somatic disorders and stigma suffered by the individuals with these disorders. Although mental disorders were the second most used category in the current study, there is evidence pointing towards under-utilization of mental disorders as primary diagnosis in DB applications, as common mental disorders are generally under-detected in primary care [17] and the patient often prefer a somatic rather than a mental diagnosis [18]. There is further considerable evidence that mental disorders are risk factors for DB award even when the primary diagnosis is a somatic condition [19], [20]. The impact of anxiety and depression on DB awards is therefore probably even greater than shown in the current study.

Mental disorder diagnoses may be also inaccurate for other reasons, which may exaggerate their impact. Common mental disorders may be used as the diagnosis in cases where continued work participation is deemed difficult or impossible, and where no other diagnosis seems suitable given the person's health status and age. Although DB are not to be awarded for social problems like unemployment, or lack of education or skills that are required in work-life, higher rates of DB have been found in communities with a difficult labor market [21], [22], among individuals with little or no education [23] and among unskilled manual workers [24]. Further, psychosocial traits and characteristics that may not be clinical conditions in themselves, such as low emotional control [25], extrovert deviant behaviour [25], problem drinking [26], lower IQ [25] and mental impairment [27] have been found to be important predictors both for DB in general and for DB awarded for mental disorder in particular. The threshold for being awarded a DB for a mental disorder has apparently been lowered in recent years [3]. Perhaps mental disorder diagnoses in some extent are being used on the DB application to secure income to individuals in a difficult life situation. However, these reasons may also apply to other diagnoses based on symptoms rather than organic findings, in particular musculoskeletal diagnoses.

Another issue with the current study regards the chosen time period, 2001 to 2003. This period was chosen because introduction of a new disability benefit scheme in 2004 affected the inflow of new DB awards the following years. The scheme was discontinued in 2010, which makes it likely that future DB figures would resemble the situation in 2001 to 2003 more than the situation in 2004 to 2009. It is, however, likely that the rate of DBs awarded for a mental disorder have increased in Norway since 2003, in concordance with the situation in several other countries within the OECD area, where mental disorders have taken over for musculoskeletal disorders as the most prevalent DB diagnoses [1]–[4], [28], [29]. If the current scenario is that a higher rate of DBs is awarded for mental disorders than in 2001–2003, the number of lost working years due to mental disorders compared to other diagnoses is likely to be even higher.

Some important limitations with the current study are also related to the underlying premise of the study; the calculation of lost working years is based on an assumption that the individual was a full-time active worker before the award of DB, followed by 100% work-life inactivity until scheduled age retirement at age 67. This is a simplified portrayal: Firstly, according to official Norwegian statistics, around 2/3 s of new DBs awarded in the period 2001–2003 were awarded for 100% disability. The remaining 1/3 were awarded for partial disability, with the majority awarded for 50 to 69% disability (25.7% of all disability benefit recipients) [30]. Secondly, the assumption of full work-life participation before the award of DB may be questioned. Attachment to work-life may vary across different diagnostic group, and employment rate among individuals with a mental disorder is particularly low [1], [6]. It is thus likely that some individuals would not have been contributing to the full time work years suggested by our analyses, had DB for a mental disorder not been awarded. Thirdly, and related, the boundaries between work ability and disability are rarely clear-cut. Most disorders will develop gradually, and increasingly affect the individual's working ability. When work disability is established, the process of DB award may take several years, even for severe mental disorders like psychosis [31]. The application period may be particular long for disorders characterized by symptoms, as the physician and patient may not initially recognize the impact of the disorder on working capacity. The decision to apply for DB may therefore be a protracted process. Fourthly, DB recipients in Norway have the opportunity to do some paid work besides the DB, and individuals who are younger or have less severe disorders may utilize this opportunity more. Individuals on DB may also return to the workforce, but this is rare [1]. In summary, these four factors (partial disability, less work participation before DB award, a long application period with work-inactivity, and some work after DB award) may be more relevant for disorders characterized by symptoms, such as mental and musculoskeletal disorders, than for more acute and severe somatic disorders. This may result in overestimation of the lost working years figures for both mental and musculoskeletal disorders.

The calculation of lost working years will also be affected by mortality before scheduled age retirement. Mental disorders, in particular schizophrenia and depression, are associated with higher mortality rates [32], [33], [34]. DB recipients in general have increased mortality rates [35], [36], but to the extent excess mortality is higher for DB recipients with mental disorders, this will inflate the estimated years of working lost in this group.

Finally, workforce composition, general economy, organization of disability benefit schemes and rehabilitation strategies vary between countries. This may affect the generalizability of the current results to other settings. However, benefit receipt at younger age among those with mental disorder diagnoses has also been found in other western countries [1], [3], [4]. The main finding of more lost working years among DB recipients with a mental disorder should thus be relevant also in other contexts.

### Why are DBs awarded at a younger age for mental disorders?

The younger age among DB recipients for mental disorders may have several explanations. For many individuals awarded DB for mental retardation, developmental disorders and psychotic disorders, their working capacity is so much reduced that they may never be in paid employment. More important than the young age among those awarded for severe mental disorders, is, however, the younger age among individuals awarded DB for a common mental disorder. Compared to somatic disorders, common mental disorders have specific characteristics that may pose greater challenges in work-life, as the main symptoms are difficulties related to behaviour, and cognitive, emotional and inter-personal functioning [15]. Further, employers may be reluctant to hire individuals with known depression, and this reluctance is more caused by perceptions of poorer work performance than expectations of future absenteeism [37]. Such perceptions may also make employers less motivated to try to keep workers with depression in work. Finally, comorbidity with both somatic and other mental health problems may increase the illness burden in individuals with common mental disorders [38], and tip the scale towards permanent work disability at an earlier age than for somatic disorders alone.

### Implications

The consequences of lost working years are probably dependent on the general health status and life situation of the individual awarded DB. For some individuals, their health may be so poor that continued work is impossible, or even dangerous. In these circumstances, the award of DB is an appropriate outcome. For others is DB an undesirable end-point. This might in particular apply to younger individuals with common mental disorders and musculoskeletal disorders. Most individuals with a mental disorder wants to work [39], and early DB award for these diagnoses may lead to severe consequences both for the individual and for wider society and the economy. For the individual, DB in general may lead to marginalization from normal social life [40], worsened health behaviour [41] and increased risk for mortality [35], including risk of suicide [42]. These adverse outcomes may be more prominent among individuals who leave the work force at a younger age, as DB award closer to retirement age may be more in concordance with the general work life participation in older age. As the size of the benefit is dependent on previous work salary, DB in young age may lead to poor economy, which also may have adverse impact on the family of the DB recipient [43]. For wider society and the economy, a high number of lost working years among working-age individuals will both provide an enormous burden on economical expenditures on disability benefits, in addition to lost tax payments. In the long run, high public expenditures on DBs may provide a great challenge for the welfare state [44]. In addition, increasing rates of DB recipients compounds to health inequalities in the population.

There are two general approaches to the prevention of work disability; treatment of the underlying health problem, and interventions aimed to prevent that the individual loses contact with the work-life. The majority of the burden from mental disorders on lost working years is caused by the massive impact from anxiety and depression. This is somewhat contrary to where official interventions to reduce work disability have previously been aimed. The majority of individuals with severe mental disorders are detected and offered treatment, usually within the specialist mental health care system [45]. A great challenge associated with common mental disorders is that they are generally under-recognized within primary health care, with the result that treatment is not being offered [46], [47]. Under-treatment is also a challenge in the context of DB award [48], [49], [50]. There are some indications that treatment of common mental disorders may decrease work impairment associated with these disorders [51], and access to evidence-based treatment for common mental disorders may be encouraged on cost-effectiveness grounds [52].

In regard to interventions developed to keep or return individuals with mental disorders to the work force, the majority of these are directed towards developmental disorders or severe mental disorders [53], i.e. Individual Placement and Support (IPS) approaches. Interventions aimed towards developmental or severe mental disorders may not be directly transferred to anxiety and depression [54], and there is currently a lack of knowledge regarding effective interventions aimed towards work disability associated with common mental disorders [55]. If DB awarded for a mental disorder is the end of an insidious process of withdrawal from working life, occupational disability resulting from the disorder may go unrecognized, and interventions may be offered too late. As long-lasting sickness absence is a key risk factor for subsequent DB [56], effort should be focused on trying to reduce the length of sickness absence, and supporting the absent individual to return to work.
